# Microfluidic model of the alternative vasculature in neuroblastoma

**DOI:** 10.1007/s44164-023-00064-x

**Published:** 2024-01-15

**Authors:** Aranzazu Villasante, Maria Jose Lopez-Martinez, Gema Quiñonero, Andrea Garcia-Lizarribar, Xiaofeng Peng, Josep Samitier

**Affiliations:** 1 Institute for Bioengineering of Catalonia (IBEC), The Barcelona Institute of Science and Technology (BIST), Barcelona, Spain; 2 Department of Electronic and Biomedical Engineering, University of Barcelona, Barcelona, Spain; 3 Biomedical Research Networking Center in Bioengineering, Biomaterials, and Nanomedicine (CIBER-BBN), Madrid, Spain

**Keywords:** Neuroblastoma, Tumor-derived endothelial cells, Vasculature, Tumor-on-a-chip, Microfluidic device, 3D tumor models

## Abstract

Neuroblastoma (NB) is a highly vascularized pediatric tumor arising from undifferentiated neural crest cells early in life, exhibiting both traditional endothelial-cell-driven vasculature and an intriguing alternative vasculature. The alternative vasculature can arise from cancer cells undergoing transdifferentiation into tumor-derived endothelial cells (TEC), a trait associated with drug resistance and tumor relapse. The lack of effective treatments targeting NB vasculature primarily arises from the challenge of establishing predictive in vitro models that faithfully replicate the alternative vasculature phenomenon. In this study, we aim to recreate the intricate vascular system of NB in an in vitro context, encompassing both types of vascularization, by developing a novel neuroblastoma-on-a-chip model. We designed a collagen I/fibrin-based hydrogel closely mirroring NB’s physiological composition and tumor stiffness. This biomaterial created a supportive environment for the viability of NB and endothelial cells. Implementing a physiological shear stress value, aligned with the observed range in arteries and capillaries, within the microfluidic chip facilitated the successful development of vessel-like structures and triggered transdifferentiation of NB cells into TECs. The vascularized neuroblastoma-on-a-chip model introduced here presents a promising and complementary strategy to animal-based research with a significant capacity for delving into NB tumor biology and vascular targeting therapy.

**Supplementary Information:**

The online version contains supplementary material available at 10.1007/s44164-023-00064-x.

## Introduction

In the last years, engineered human tissues designed for regenerative therapies have taken on a miniature form known as organs-on-a-chip (OOAC). These OOACs are compact plastic devices, no larger than a thumb, featuring microchannels for cultivating diverse human cell types and small chambers filled with culture medium. Within this setup, human cells are cultured in a controlled microenvironment closely mirroring organ functions, enabling the exploration of biomedical phenomena [1].

An impactful application of this technology, reshaping cancer research and the pharmaceutical sector, is the creation of tumors-on-a-chip (TOAC). The key advantage of this technology lies in the intricate design of each TOAC as a comprehensive microsystem, replicating the cellular composition and microarchitecture of native tumors while emulating specific functions in a scaled-down 3D format. This innovation expedites the translation of therapeutic discoveries to patients in need. Beyond their predictive capabilities favoring precision medicine, TOACs offer a remarkably adaptable platform utilizing minimal cell quantities and microlitre volumes of reagents, all in a cost-effective and time-efficient manner. Furthermore, TOACs seamlessly integrate with high-throughput drug screening platforms [2–4].

Neuroblastoma (NB) is one type of solid tumor that could be miniaturized by using TOAC technology, and for which we need better preclinical models and a cure. NB is a malignant tumor of the neural crest cells that give rise to the sympathetic nervous system. It is a rare cancer and the most common extracranial solid tumor of childhood diagnosed in the first year of life. It frequently arises in the adrenal medulla, although the tumor’s location can be anywhere in the sympathetic nervous system (abdominal origin in 80% of cases). MYCN oncogene is frequently amplified in high-risk NB, linked to an undifferentiated phenotype and poor prognosis. NB is also a highly angiogenic tumor [5–7].

Tumor vascularization is crucial for regulating growth, survival, and metastasis in solid tumors. Tumor vessels can be formed through physiological mechanisms like angiogenesis (sprouting and intussusceptive) and vasculogenesis. Initially, sprouting angiogenesis generates new blood vessels from pre-existing ones, while intussusceptive angiogenesis involves splitting vessels through intra-luminal tissue pillars. Tumors also utilize embryonic vasculogenesis, where vascular precursors differentiate into endothelial cells [8, 9]. Tumor vascularization strategies also encompass cancer-specific methods like vessel co-option, integrating healthy organ vessels into the tumor, as well as alternative mechanisms such as vasculogenic mimicry and endothelial transdifferentiation. In vasculogenic mimicry, tumor cells align and form CD31-negative, periodic acid-Schiff (PAS)-positive vessel-like structures. Endothelial transdifferentiation enables cancer cells to acquire endothelial traits, including CD31 expression, resulting in tumor-derived endothelial cells (TEC) [8, 9]. Cancer-specific vessel formation strategies are likely implicated in chemoresistance across various tumors [10, 11]^.^

In the past two decades, numerous studies have explored the correlation between tumor advancement and angiogenesis in NB using both in vitro and in vivo experimental models. Consequently, targeting vasculature and suppressing vasculature-related factors like VEGF has been postulated as a potential approach for treating NB [12, 13]. VEGF plays a crucial role in neuroblastoma by promoting endothelial cell growth, angiogenesis, and migration. Numerous experimental antiangiogenic treatments, including VEGF inhibitors and antibodies against VEGF receptors, strive to block the VEGF signaling pathway, thus hindering the formation of tumor blood vessels. Despite the effort, antiangiogenic strategies effective in preclinical models have not translated to improved patient survival in clinical trials [12].

NB also employs one of the alternative vascularization mechanisms previously mentioned, where NB cells transform into TEC-expressing CD31 and MYCN amplification markers [14]. Current antiangiogenic approaches primarily focus on angiogenesis-generated vasculature, overlooking alternative mechanisms that could cause the clinical setbacks observed [9]. Hence, comprehending TEC biology is crucial for designing effective therapies in NB. However, the lack of effective treatments for targeting TEC and the alternative vasculature in neuroblastoma is primarily due to the challenge of creating accurate predictive in vitro models replicating this phenomenon. Establishing representative research models of TEC behavior is essential for advancing understanding and developing targeted therapies for neuroblastoma.

Thus, we proposed creating an NB-on-a-chip model encompassing both the “traditional” vascularization driven by angiogenesis and the alternative vasculature generated by transdifferentiation observed in patients. To achieve this, we replicated the physiological stiffness of NB by fabricating a soft collagen-based biomaterial with Young’s modulus of approximately 1 kPa. This biomaterial, designed to mimic the natural extracellular matrix mechanical stimulus, supported the proliferation and viability of NB and endothelial cells. Various combinations of microenvironmental factors were employed to culture NB cells; yet, a static environment failed to trigger vasculature formation or transdifferentiation. However, the introduction of shear stress through the microfluidic device proved pivotal. This induced the expression of CD31 in NB cells, leading to their transformation into tumor-derived endothelial cells and the consequent generation of alternative vasculature. Our innovative NB-on-a-chip model is the first tool for evaluating different vasculature responses to drugs in a high-throughput, cost-effective, and ethically sound platform, serving as a preliminary step before assessing treatment effectiveness in more intricate models such as murine models and actual patients.

## Materials and methods

### Hydrogel preparation and characterization

FX3 and FX5 formulations are both collagen I/fibrin-based hydrogels. They were fabricated using rat tail collagen I (Corning, # 354249), fibrinogen from bovine blood plasma (Sigma #F8630), aprotinin (Sigma #AG106), M199 medium (Sigma # M9163), and HEPES (Gibco #15630080). Briefly, materials were fabricated as follows: thrombin was reconstituted at 100 U/mL, fibrinogen at 50 mg/mL, and aprotinin at 1 mg/mL using sterile PBS. Additionally, 1.5 M NaHCo3 and 1 M HEPES solutions were prepared in sterile distilled water. To fill up 7 chips, 100 μL of collagen I/fibrin-based hydrogels were prepared using rat tail collagen I (2 μg/μL), fibrinogen from bovine blood plasma (10 μg/μL), aprotinin (80 ng/μL), M199 medium, and HEPES and pH adjusted to 7.4 with 1.5 M NaHCO3. The cross-linking process employed 0.00125 U/μL of thrombin (Sigma # T4648) for FX3 and 0.0025 U/μL for FX5.

#### Mechanical properties

Uniaxial compression tests of materials were performed using a Zwick Z0.5 TN instrument (Zwick-Roell, Germany) with a 5 N load cell. Cylindrical samples were cut using a 6-mm diameter biopsy punch. Absolute diameters and heights were measured before the experiment. Samples were tested at room temperature up to 30% final strain (deformation), using the following parameters: 0.1 mN preload force and 20% min^−1^ strain rate. Stress–strain graphs were obtained from load–deformation measurements. Values for the compressive modulus were calculated from the slope of the linear region corresponding to 10–20% strain. Three samples were prepared for each material, and measurements were performed in triplicate.

#### Fluid uptake of the hydrogels

Dried samples were weighed (Wd) and immersed in distilled water at 37°C for different periods (2 h, 3, 5, 7, and 10 days). At each time point, specimens were removed from distilled water, and the ability of the scaffold structure to absorb water was measured. At each time point, the samples were removed from water and weighed (Ww). The water uptake was calculated as Fluid uptake (%) = (Ww–Wd)/Wd × 100. Each sample was measured in triplicate.

#### Hydrogel degradation

Dried samples were weighed (Wd) and immersed in distilled water at 37°C in a humid atmosphere for timed intervals (2 h, 3, 5, 7, and 10 days). At each time point, specimens were removed from distilled water, air-dried for 24 h, and weighed (Wa). The weight loss was calculated as weight loss (%) = (Wd–Wa)/Wd × 100. Each sample was measured in triplicate.

#### Scanning electron microscopy (SEM)

For 1 h, the hydrogels were fixed with formalin 10% neutral buffered (Sigma #HT5011). After three 10-min washes in PBS and two 10-min washes in Milli-Q water, hydrogels were dehydrated in ethanol series: 30%, 50%, 70%, 90%, and two 100% ethanol washes for 5 min each. Finally, the gels were dried using the critical point technique, mounted on SEM sample stages using double-sided carbon tape, and sputter-coated with gold. Samples were visualized using the SEM microscope JSM7001F (Jeol), and the acceleration voltage was 5.0 kV.

#### Porosity measurement

The porosity of the hydrogels was assessed using the scanning electron microscopy (SEM) method [15]. This method was adapted from Anguiano et. al. [16]. Image processing techniques were employed to determine the porosity of the biomaterials. SEM images were analyzed in ImageJ software, where a binary mask was generated to highlight the biomaterial strands selectively. By applying thresholding and particle analysis, individual pores within the biomaterial structure were identified and quantified. The porosity was calculated using the formula:

$$\mathrm{Porosity}\;(\%)\;=\;(\mathrm{total}\;\mathrm{pore}\;\mathrm{area}/\mathrm{total}\;\mathrm{image}\;\mathrm{area})\times100$$

### Cell culture

#### Neuroblastoma cells

SK-N-BE (2) (from the American Type Culture Collection, ATCC) was cultured according to the manufacturer’s specifications in RPMI medium supplemented with 10% (v/v) FBS and 1% penicillin/streptomycin. Cells were cultured at 37°C and 5% CO_2_ in a humidified incubator.

#### Human umbilical vein endothelial cells (HUVEC)

Human umbilical vein endothelial cells (HUVEC) were purchased from Lonza and cultured according to the manufacturer’s specifications. HUVEC cells were cultured in HUVEC expansion medium EBM-2 basal medium (Lonza CC-3156) supplemented with EGM-2 BulletKit (Lonza CC-3156 & CC-4176) at 37°C in a humidified incubator at 5% CO_2_.

#### Live-dead assay

Samples were incubated in RPMI medium containing 2μM calcein and 4μM of ethidium homodimer-1 for 30 min at 37°C, 5% CO_2_, as indicated by the manufacturer’s protocol (LIVE/DEAD® Viability/Cytotoxicity Kit, Molecular Probes). Samples were imaged with a fluorescence microscope (Olympus IX81 light microscope, Center Valley, PA).

### Quantitative real-time PCR (qRT-PCR)

Total RNA from cells was obtained using Trizol (Life Technologies), following the manufacturer’s instructions. RNA preparations were treated with “Ready-to-go you-prime first-strand beads” (GE Healthcare) to obtain cDNA. Quantitative real-time PCR was performed using PowerUp SYBR Green Master Mix (Applied Biosystems #A25742). mRNA expression levels were quantified by applying the ΔCt method, ΔCt = (Ct of the gene of interest - Ct of GAPDH). PECAM, CD34, and MYCN primers were obtained from the PrimerBank database (http://pga.mgh.harvard.edu/primerbank/); see Table 1.

**Table 1:** List of primers used for quantitative real-time PCR in this study

Gene	NCBI gene ID	PrimerBank ID	Sequence
MYCN	4613	62750358c1	ACCCGGACGAAGATGACTTCT
			CAGCTCGTTCTCAAGCAGCAT
PECAM	5175	313760623c1	AACAGTGTTGACATGAAGAGCC
			TGTAAAACAGCACGTCATCCTT
CD34	947	68342037c1	CTACAACACCTAGTACCCTTGGA
			GGTGAACACTGTGCTGATTACA
GAPDH	2597	83641890b1	AAGGTGAAGGTCGGAGTCAAC
			GGGGTCATTGATGGCAACAATA

### Immunofluorescence (IF)

Cells in 2D were fixed with formalin 10% neutral buffered (Sigma #HT5011) for 15 min at room temperature. Cells in 3D were fixed for 30 min. Fixed samples were permeabilized for 15 min in 2D and 30 min in 3D with PBS containing 0.2% Triton X-100 and blocked for 1 h with 5% BSA in PBST (PBS+ 0.1% Tween 20). Then, samples were incubated with primary antibodies for MYCN (MYCN-rabbit: dilution 1:100; Abcam, ab198912) and CD31 (CD31-mouse dilution 1:50, DAKO, #M0823) diluted in antibody diluent (Dako, S3022) in a humid chamber overnight at 4°C. A day later, the samples were washed (3 times, 5 min each) with PBST and incubated with the appropriate secondary antibodies (ThermoFisher; Goat anti-rabbit-Alexa Fluor 488 # A-11008; goat anti-mouse-Alexa Fluor 647 #A-21235) diluted 1:500 in antibody diluent at room temperature, in a humid dark chamber for 1 h. Secondary antibodies made in mouse and rabbit were co-incubated at the same time. Samples were incubated with Hoechst (dilution 1:5000) for 15 min at room temperature and imaged using the Nikon Eclipse Ti2 or Zeiss LSM 800 microscopes.

### Microfluidic device and perfusion setup

**Fig. 1:**
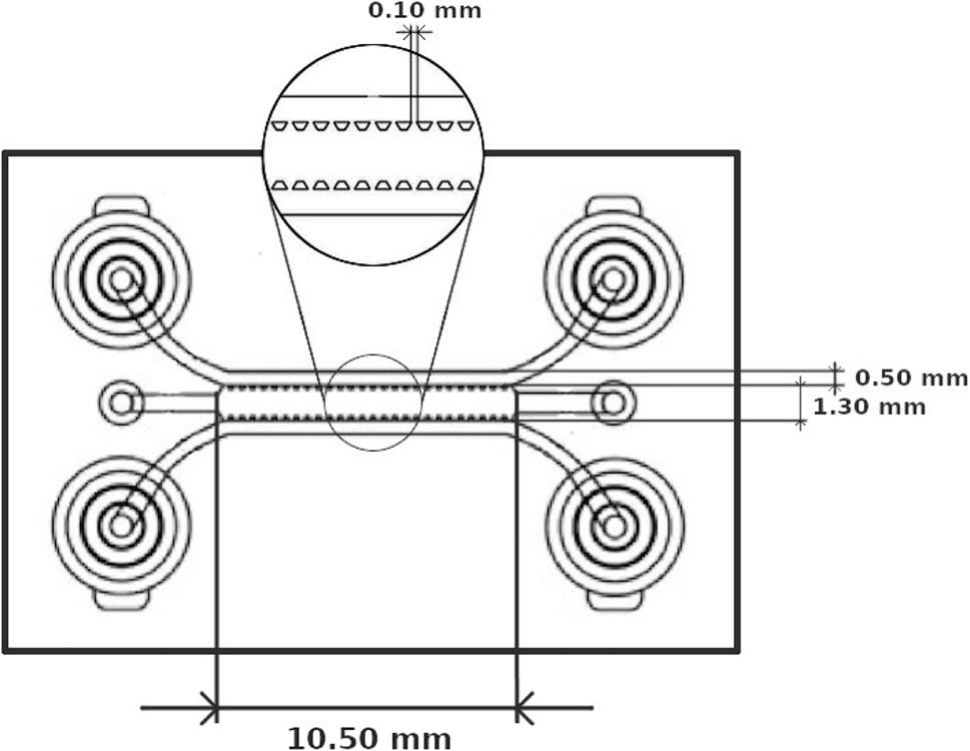
idenTx3 microfluidic chip dimensions. The schematic illustrates the design of the idenTx3 microfluidic chip, featuring three distinct channels. The central channel hosts a 3D gel region, flanked by two parallel media channels. This representation is adapted from the original design available at aimbiotech.com/product/identx-3-chip

idenTx 3 Chips (AIM Biotech) were assembled in an idenTx 9 Plate for the indicated experiments. LUC-1 luer (AIM Biotech). Chip architecture and dimensions are depicted in Fig. 1.

The introduction of cells adhered to a standardized protocol from the manufacturer. In summary, cells were integrated into the hydrogel mixture and introduced into the central chamber of the microfluidic chip. To prevent biomaterial overflow into media channels, a critical volume limit of 13 μl for the biomaterial/cells mixture was carefully taken using a micropipette to avoid bubbles. The central chamber was initially filled with 6.5 μl of the mixture from one inlet, stopping near the end of the posts, and subsequently filled from the other inlet with 6.5 μl until the mixture fronts merged. Reservoirs were filled with 200μl/reservoir of culture medium, and chips were cultured at 37°C and 5% CO_2_ in a humidified incubator for 24h or 48h under static conditions.

Perfusion setup: male luer integral 1/16 connectors (Cole-Parmer #45518-07) were used to connect Tygon tubes (Tygon AAD04133; inner diameter = 1.27mm, outer diameter = 2.28 mm) to chip inlets and outlets. Tygon tubes were connected to the peristaltic pump tubing (Tygon LMT-55 #070534-04-ND) through a small piece of Tygon tube of a 2-mm inner diameter (Tygon # ACSF1S1502-C). The recirculating circuit was achieved by attaching the other extreme of Tygon tubes to a reservoir fabricated with a 50-mL Falcon. The falcon tube was also used as a bubble trap system. The culture medium was pumped using a high-precision IPC-N 8 peristaltic pump (Ismatec) and constantly perfused for 6 days to get a wall shear stress of 36 dynes/cm^2^. The idenTx 9 plates containing the devices were placed inside a humidified incubator at 37°C and 5% CO_2_.

### Diffusion analyses

#### Ink diffusion assay

To evaluate the macroscopic fluidic behavior, structural integrity, and homogeneity of the hydrogel, an ink diffusion assay was conducted. A commercial pen ink (Pilot Hi-Tecpoint V7) was utilized for this assay. One hundred microliters of ink were carefully pipetted into the top reservoirs of the microfluidic chip, allowing it to flow through one of the channels until reaching the other channel at the bottom side. This approach facilitated the observation of overall structural features and fluidic behavior at a larger scale.

#### FITC–dextran diffusion assay

For microscale investigations into substance diffusion within the hydrogel, fluorescein isothiocyanate–dextran (FITC–dextran), with a molecular weight of 70 kDa (Sigma, 46945-100MG-F), was selected as a representative tracer for substances of moderate size. This choice aimed to simulate the behavior of molecules such as nutrients or potential therapeutic agents. The FITC–dextran solution was introduced through the top channel, establishing a diffusion gradient similar to the ink assay.

### Histological studies

Microfluidic devices were opened, and tumor tissue constructs were washed in PBS and fixed in 10% formalin overnight at 4°. Then, the samples were processed for histological analysis; they were dehydrated in graded ethanol washes and embedded in paraffin. Serial sections (5 μm thick) were mounted on glass slides and stained using routine hematoxylin/eosin procedures.

### Statistical analysis

Statistical significance was assessed using the two-tailed Student’s *t*-test, comparing the means of two groups for various analyses. The significance levels were set at *p < 0.05, **p < 0.01, and ***p < 0.001, with “ns” indicating non-significance.

## Results

**Fig. 2:**
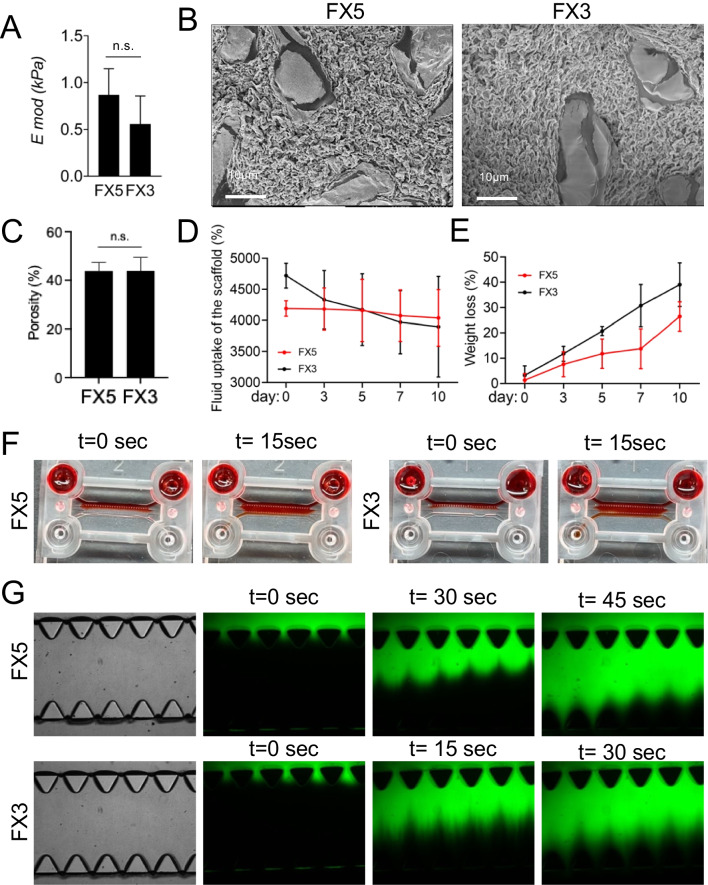
Characterization of collagen/fibrin-based hydrogels. **A** Equilibrium modulus (E mod) of the indicated formulations FX5 (*n* = 3) and FX3 (*n* = 3) recapitulating native stiffness of neuroblastoma tumors. **B** Scanning electron microscopy (SEM) images of the FX5 (*n* = 3) and FX3 (**n** = 3) formulations. **C** Porosity evaluation for the specified materials (*n* = 3). **D** Fluid uptake of the hydrogels in water (*n* = 3 per condition and time point). **E** Degradation of the FX5 and FX3 hydrogels (*n* = 3 per condition and time point). **F** Homogeneous diffusion of red ink through FX5 and FX3 hydrogels, illustrated at the initial time point (*t* = 0 s) and the final time point (*t* = 15 s) (*n* = 3; seconds, sec). **G** Fluorescence microscopy images displaying the diffusion of FITC-conjugated dextran into the indicated biomaterials within the gel region of the chip at specified time points (*n* = 3; seconds, sec)

We first aimed to engineer a biomaterial that emulates the intrinsic stiffness observed in tumors from patients to replicate the physiological microenvironment inherent to NB more accurately. As a soft tumor, neuroblastoma exhibits a characteristic low Young’s modulus (*E*_mod_) of *E*_mod_ = 0.79 ± 0.20 kPa [17]. We previously developed and validated a non-physiological stiffness collagen I/fibrin-based hydrogel to support vascularization and endothelial transdifferentiation of NB cells [18]. Subsequently, we opted to finetune this biomaterial by modulating the concentration of the thrombin enzyme. This modification resulted in two distinct and consistent formulations (FX) with stiffness values that were not statistically different, falling within the range of natural NB stiffness. These formulations are named FX5 (*E*_mod_ = 0.87 ± 0.28 kPa) and FX3 (*E*_mod_ = 0.56 ± 0.30 kPa) **(**Fig. 2A). Porosity studies also revealed no statistical differences, with both biomaterials having approximately 50% porosity (FX5 porosity = 56.2 ± 3.5; FX3 porosity = 56.1 ± 5.6) (Fig. 2B–C). The swelling percentage of the scaffolds over time in both formulations was measured to investigate their fluid absorption behavior (Fig. 2D). Both materials did not show significant differences regarding swelling behavior over time. The study of the percentage of weight loss over time demonstrated that the FX5 material is more stable in liquids than the FX3; the FX5 can remain almost intact until day 7 while the FX3 is partially degraded (Fig. 2E). Based on previous experience, at least 7 days of cell culturing are necessary to obtain biomimetic responses in a bioengineered cancer model [19].

Gelation inside the chip was also analyzed, and time-dependent diffusion of ink and FITC–dextran into the hydrogel matrices was monitored. Both biomaterials were perfectly gelled within the microfluidic device’s central chamber without interfering with culture media perfusion through the lateral microchannels (Fig. 2F–G).

We also noted homogeneous ink diffusion (Fig. 2F) and FITC–dextran penetration (Fig. 2G) throughout the entire hydrogel within the microfluidic chip for both formulations. Notably, the diffusion of FITC–dextran through the FX3 hydrogel occurred at a faster rate compared to FX5 (Fig. 2G). These findings collectively suggest that an in vitro tumor model could facilitate nutrient supply to the cells through diffusion.

We then conducted 2D static experiments by seeding cells on top of the biomaterials before introducing the cells inside the chip device (3D cultures). This initial approach allowed us to assess the biocompatibility and cytotoxicity of the formulations under conditions where cells had complete access to oxygen and nutrients. The subsequent transition to the 3D microfluidic chip analysis was guided by these preliminary findings, ensuring that any observed effects could be confidently attributed to the specific properties of the 3D niche rather than the potential toxic effects of the biomaterial.

**Fig. 3:**
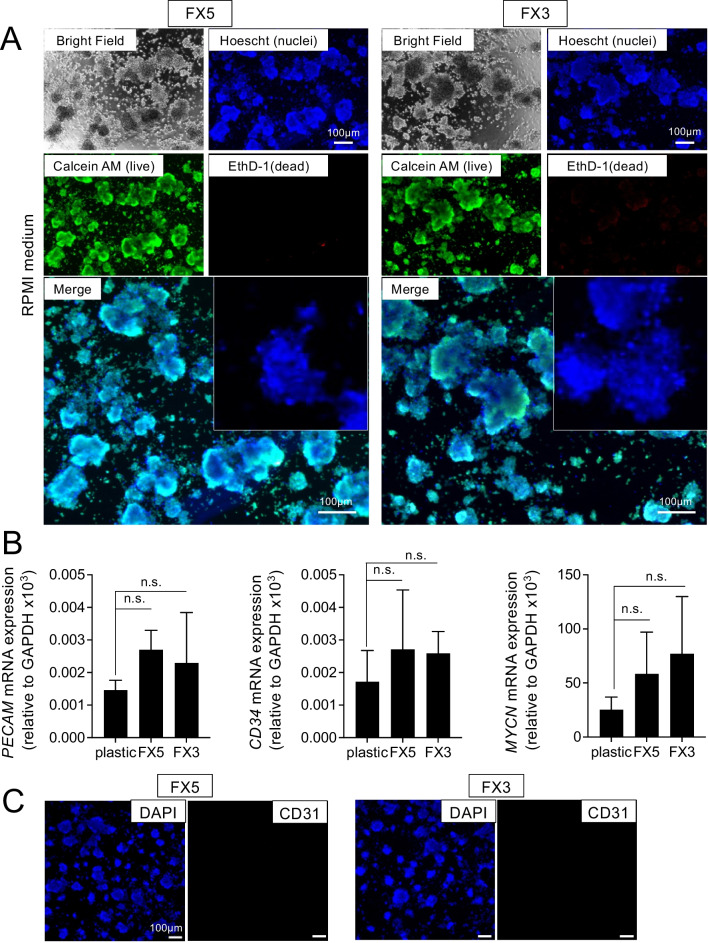
Biocompatibility and endothelial transdifferentiation of neuroblastoma cells on collagen/fibrin-based hydrogel-coated 2D substrates. **A** Live/dead cell viability assay of neuroblastoma cells (SKN-BE (2)) cultured on FX5 or FX3 substrates. Top left corner: representative bright-field images for each set. The cells were cultured in RPMI for 7 days and then stained with the calcein AM (live cells; green), ethidium homodimer 1 (EthD-1; dead cells, red), and Hoechst 33342 (nuclei; blue) (*n* = 5). Merge images of the three stains are shown at the bottom, along with a zoomed image of the nuclei stain Hoechst 33342. **B** PECAM, CD34, and MYCN mRNA levels in SKN-BE (2) cultured on the indicated substrates in RPMI culture medium for 7 days. Fold change was calculated by first normalizing to GAPDH levels in the individual samples and then to the corresponding levels in cells cultured in plastic culture dishes. Data are shown as average ±SD (*n* = 3); n.s., not significant. **C** Representative fluorescence images of neuroblastoma cells cultured on FX5 and FX3 materials and stained for CD31 (magenta) (*n* = 3); nuclei are stained with DAPI (blue)

We cultured neuroblastoma cells in the model system in RPMI culture medium. Live/dead assays showed both materials’ biocompatibility and null cytotoxicity for at least 7 days (Fig. 3A). This set of experiments could also give important preliminary information about the biomaterials’ support for enhancing the endothelial phenotype (CD31^+^ cells). In this case, neither material was optimum for enhancing the expression of the endothelial markers CD31/PECAM (Fig. 3B, C) or CD34 (Fig. 3B) and, therefore, endothelial transdifferentiation of monocultures of neuroblastoma cells. The study of MYCN expression in the neuroblastoma cells revealed a tendency to increase when cultured on biomaterials compared to plastic plates, although it was not statistically significant (Fig. 3B).

**Fig. 4:**
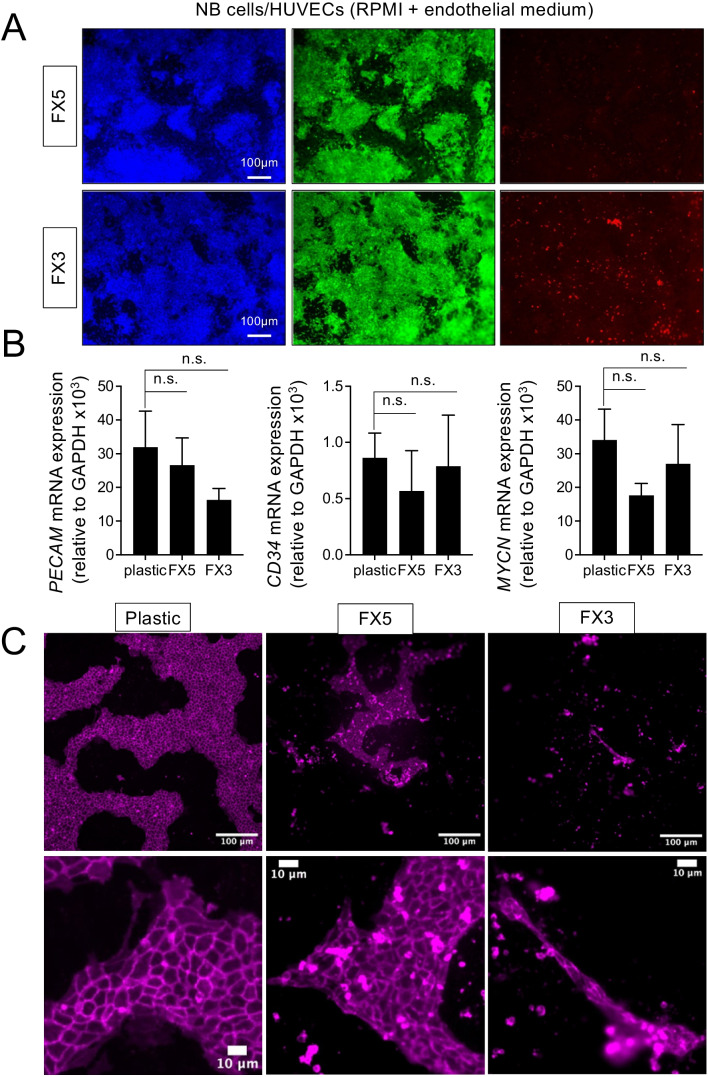
Studies of cell survival and endothelial cell culture compatibility of co-cultures of neuroblastoma and endothelial cells on collagen/fibrin-based hydrogel-coated 2D substrates. **A** Representative live/dead staining images of neuroblastoma cells in co-cultures with human umbilical vein endothelial cells (HUVEC) with a mix of RPMI/Endothelial (EGM) medium at day 7 (*n* = 4). Calcein staining (green-live cells), ethidium homodimer-1 staining (red-dead cells), nuclei (Hoechst 33342). **B** qRT-PCR of *PECAM*, *CD34*, and *MYCN* expression in the co-cultures cultured in RPMI/EGM during 7 days in the indicated substrates (plastic and FX5 and FX3 hydrogels) (*n* = 3). Relative endogenous expression of genes was normalized to *GAPDH*, and error bars represent standard deviations of relative expression. n.s., not significant. **C** Analysis of endothelial cell culturing compatibility on the indicated substrates. Representative fluorescence images of neuroblastoma and HUVEC co-cultures stained for CD31 (magenta) (*n* = 3)

We also assessed the capability of the materials in the preliminary 2D studies to support cell survival in general and endothelial cell maintenance in particular in co-cultures of NB cells and HUVECs using an RPMI and endothelial medium (EGM) mix (1:1) for 7 days. The FX5 biomaterial showed a better capacity to sustain the co-culture alive than the FX3 (Fig. 4A). Interestingly, live/dead analyses of NB cells in monoculture treated with the RPMI/EGM mix resulted in the condition with higher mortality compared to the other treatments (Supp. Figure 1A).

Neither biomaterial significantly affected PECAM, CD34, and MYCN gene expression levels (Fig. 4B), but the FX5 material supported the formation and survival of grouped cells expressing CD31 more effectively than FX3 (Fig. 4C).

Based on the biocompatibility 2D results, we selected the FX5 material, the co-culture of NB/HUVEC cells, and the RPMI/EGM medium as the most suitable conditions for further studies in the chip.

After confirming the non-toxic nature of the biomaterial, our assessment extended to overall cell viability and its support for endothelial cells within the 3D context of the NB-on-a-chip. Initial experiments involved determining the optimal cell density for culture, evaluating the survival of the co-culture over time under static conditions. In the absence of perfusion during these conditions, we took measures to ensure that any observed effects could be reliably attributed to the specific properties of the 3D niche.

**Fig. 5:**
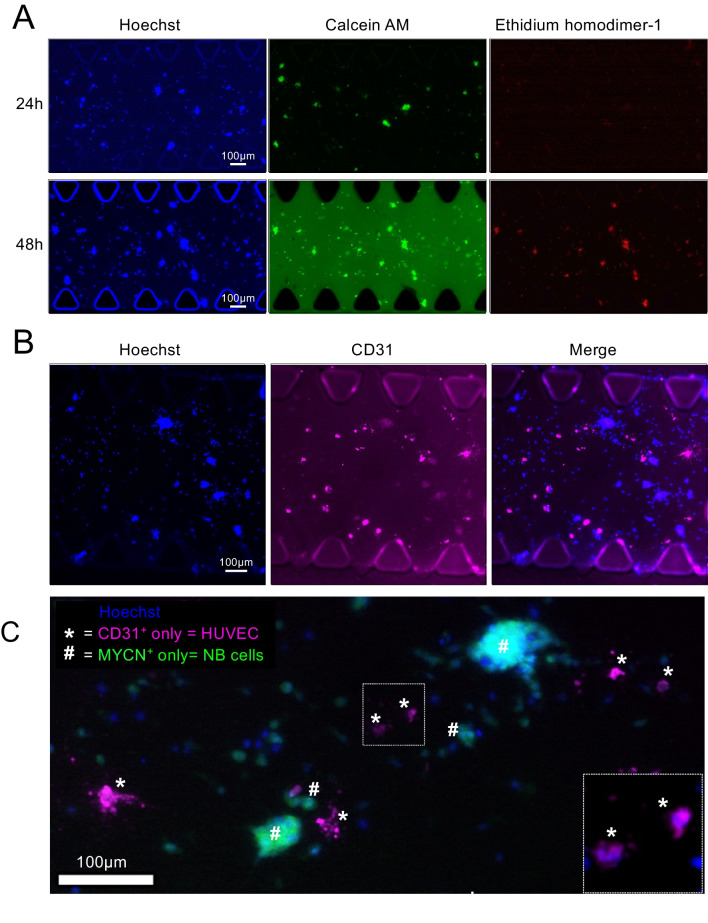
Analysis of cell survival, endothelial cell culturing compatibility and transdifferentiation of 3-dimensional neuroblastoma/HUVEC co-cultures in a microfluidic device under static conditions. **A** Live/dead fluorescence images of neuroblastoma and HUVEC co-cultures seeded in the 3-dimensional FX5 hydrogel loaded in the central chamber of the chip for 24 and 48 h; 1000 cells/chip co-cultures (*n* = 5 per time point). Hoechst staining (Hoechst 33342, nuclei; blue), calcein staining (green-live cells), ethidium homodimer-1 staining (red-dead cells). **B** Representative fluorescence images of neuroblastoma and HUVEC co-cultures-stained for CD31 (endothelial marker; magenta) (**n** = 3); nuclei are stained with Hoechst 33342 (blue). **C** Fluorescence image of the cells within the FX5 hydrogel in the central chamber of the chip double-stained for MYCN (neuroblastoma marker; green), CD31 (endothelial marker; magenta), and Hoechst staining (Hoechst 33342, nuclei; blue) (*n* = 3)

We assayed 500,1000, 2000, and 3000 cells/chip (data not shown). The chip contained four cell culture media reservoirs, a cover for evaporation control, and the medium was changed daily. However, none of the conditions survived more than 48h, but 1000 cells/chip was the best cell density and survival condition. One thousand cells/chip co-cultures were completely alive at 24h after seeding. However, they showed high cell death at 48h (Fig. 5A**;** Supplementary Figure 2).

The FX5 biomaterial facilitated the development and persistence of clustered cells expressing CD31 in a 3D configuration (Fig. 5B), consistent with observations from preliminary 2D experiments. This investigation was specifically focused on the survival of any endothelial cells—HUVEC or TEC—by examining the expression and distribution of the endothelial CD31 marker. Notably, at 24 h, we observed the initiation of sprouting, evolving further by 48 h (Supplementary Figure 2C). The origin of CD31 expression could be attributed to NB cells, exclusively HUVECs, or a combination of both within the co-culture system. To address this uncertainty, we conducted co-staining for CD31 (an endothelial marker for HUVECs and TECs) and MYCN (an NB-specific marker) within the tissue (Fig. 5C). Intriguingly, no co-localization of both signals was observed. Our interpretation is that the clustered cells expressing CD31 in 3D under static conditions were likely mono-cellular aggregates formed predominantly by HUVEC cells.

Even though the hydrogel was designed to facilitate nutrient diffusion, we argued that the higher rate of cell death observed at 48 hours within the chip might have been caused by insufficient nutrient availability. Perfusion could avoid this issue by favoring higher nutrient availability through a higher renewal rate. During perfusion culture, the cells seeded can sense the shear stress exerted by the fluid flow. Since shear stress has been shown as a pivotal microenvironment factor to lead to vasculature formation, we hypothesized that it would be necessary to include it in the system to favor cell survival and achieve vascularization. Shear stress varies based on the type of blood vessel, ranging from 10 to 60 dynes/cm^2^ in arteries, 1 to 10 dynes/cm^2^ in veins, and 3 to 95 dynes/cm^2^ in capillaries [20, 21]. We selected a wall shear stress of 36 dynes/cm^2^ (143 μl/min) as the physiological value matching the arteries and capillaries' shear stress range.

**Fig. 6:**
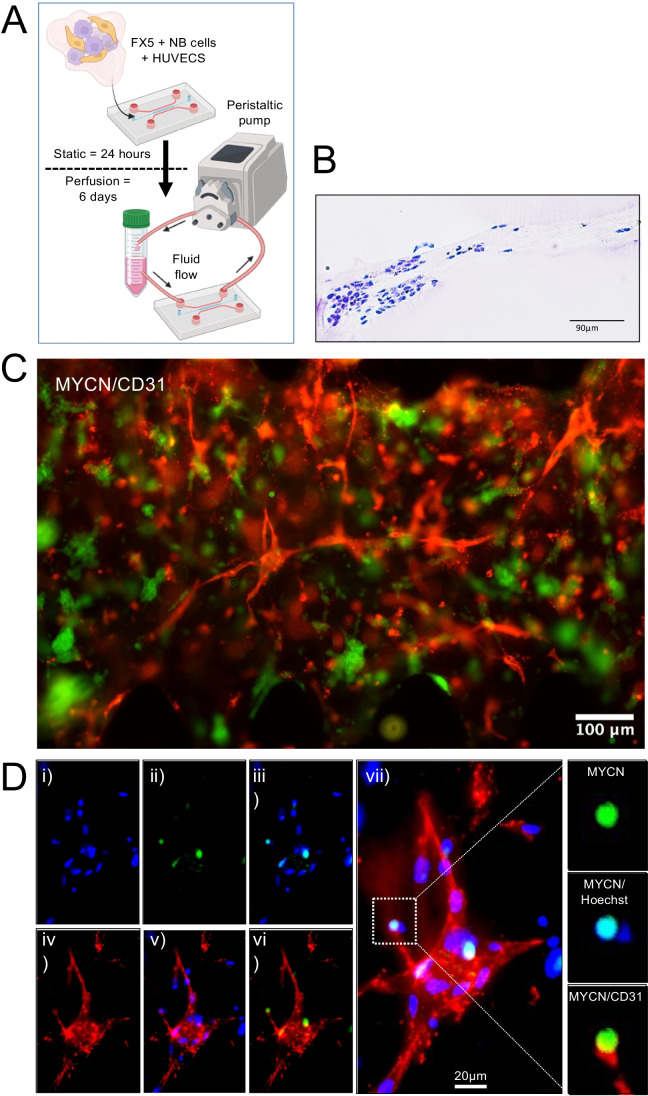
Analysis of endothelial cell transdifferentiation in a perfused neuroblastoma-on-a-chip. **A** Schematic depiction of the experimental protocol to build the vascularized neuroblastoma-on-a-chip. 1000 cells/chip of neuroblastoma/HUVEC co-cultures are embedded in the FX5 hydrogel, placed in the central chamber of the microfluidic chip, and cultured for 24 h with a mix of RPMI/EGM culture medium (1:1). The system is perfused applying a wall shear stress of 36 dynes/cm^2^ (143 μl/min) for 6 days using a peristaltic pump. The diagram illustrates the dual purpose of a Falcon tube, serving as both a medium reservoir and a bubble trap. Created with BioRender.com. **B** Representative image of paraffin-embedded tissue sections stained with hematoxylin and eosin (H&E) obtained on day 7 after cell seeding and under perfusion conditions (*n* = 3). **C** Fluorescent image of the chip seeded with NB cells and HUVEC within the FX5 hydrogel in the central chamber and cultured in RPMI/endothelial (EGM) mix medium under flow perfusion conditions for 6 days. Cells were double-stained for MYCN (neuroblastoma marker; green) and CD31 (endothelial marker; red) (*n* = 6). **D** Image showing tumor-derived endothelial cells (TEC) in the vascularized tissue generated in the microfluidic device. (i) Hoechst 33342 staining (blue, nuclei), (ii) MYCN staining (neuroblastoma marker, green); (iii) image showing co-localization of MYCN in the nuclei of neuroblastoma cells; (iv) CD31 (endothelial marker; red); (v) Hoechst 33342/CD31 double staining; (vi) MYC/CD31 double staining showing neuroblastoma cells expressing the endothelial marker indicating the presence of TEC in the tissue; (vii) merge (Hoechst 33342-blue, MYCN-green, CD31-red). Inset showing in detail an NB cell, co-stained for MYCN (NB-nuclear marker) and Hoechst 33342 (nuclear marker), expressing the surface marker CD31 (MYCN/CD31 image)

Consequently, a co-culture of NB cells and HUVEC were seeded within the FX5 hydrogel and cultured in RPMI/EGM mix medium for 24h, and then perfusion was applied for 6 days **(**Fig. 6A). We confirmed tissue formation by histological analysis (Fig. 6B). Endothelial tissue was identified when 36 dynes/cm^2^ were applied to the system by CD31 endothelial marker staining (Fig. 6C). Finally, we also demonstrated the formation of the alternative vasculature in the tissue by the identification of tumor-derived endothelial cells, which expressed both endothelial (CD31, cell membrane) and neuroblastoma (MYCN, nuclear) markers at the same time (Fig. 6D).

## Discussion

Cancer cells adapt their features to their surrounding microenvironment to survive and proliferate. Thus, cancer cells seeded in traditional plastic cultures modify their gene/protein expression signature, such as focal adhesion genes, metabolic profile, and differentiation response, to survive in this new scenario outside the body. If adaptation does not happen, cancer cells can even die. Moreover, those changes in cancer cell features lead to different sensitivities to therapeutic options and drug responses. Cancer cells cultured in 2D often have little resistance to drugs [22–24]. For example, cancer cells are most sensitive to pro-apoptotic treatments when culture in plastic dishes may not be translatable to the clinic [25]. Importantly, 2D cancer models lack cell heterogeneity observed in clinical samples in patients. Tumors are not merely composed of cancer cells but also interact with various other cell types and stromal components. Adaptation to a plastic plate produces the elimination of subpopulations of cells involved in survival in a harsh environment that is the human body, for example, cells controlling tumor vasculature formation or cancer stem cell niche [26, 27]. Those subpopulations represent only a tiny percentage within a tumor cell mass and its microenvironment, but they are critical for tumor development. Considering this, we can argue that drugs that affect cancer cells in plastic culture models may not act over essential subpopulations of cells present in native tumors but are not represented in the in vitro model. Thus, it seems critical to recapitulate in vitro those populations of cells, such as TEC, that have a relevant role in tumor maintenance and drug resistance to assess a specific treatment against them. Bioengineering tools are being applied to develop complex microenvironments–based biomaterials coupled to bioreactors to recapitulate tumor behavior and better predict therapeutic responses [28].

Developing biomaterials that accurately mimic the tumor microenvironment is a significant challenge in cancer engineering research. Selecting an appropriate biomaterial is crucial, as it can profoundly influence cell behavior and the presence/absence of tumor cell subpopulations and, therefore, heterogeneity and therapeutic responses.

In this study, we addressed this challenge by designing biomaterials with composition and stiffness similar to native NB tumors, aiming to create a more physiologically relevant in vitro model. Among the biomaterial formulations tested, FX5 emerged as the most promising candidate. Its ability to support viable monocultures of NB and co-cultures of NB cells and HUVECs underscores its compatibility with both cell types.

Culture media used is another critical aspect to obtain the desired cell response. Besides endothelial cell maintenance, EGM medium is also used for differentiating mesenchymal stem cells into the endothelial lineage [29]. NB cells exhibit undifferentiated traits and express stemness-related markers. So, we hypothesized that culturing the NB cells with EGM could induce vasculature formation. However, NB cells in monoculture within FX5 but with endothelial culture medium (EGM) produced high cell death. The alive cells displayed planar morphology and lost their characteristic growing in aggregates, similar to ES/iPS when differentiated. We can argue that the EGM medium could initially trigger the (trans)differentiation process, but this insult seemed insufficient to obtain TEC, and finally, cells died.

The choice of the cell type to include in the model system is also crucial. In this study, we used HUVECs. The selection of HUVEC is grounded in the extensive evidence demonstrating their capability to form microvessels in various organ-on-a-chip models and bioengineered-based systems [30–32]. Notably, studies by Schonherr-Hellec et al. [33]and Uwamori et al. [34] have utilized HUVECs for constructing 3D-printed scaffolds and microvascular networks within a microfluidic device, respectively. In the study by Schonherr-Hellec et al., HUVECs were employed to develop a 3D-printed scaffold supporting the growth of mature microvascular networks both in vitro and in vivo, demonstrating the versatility of HUVECs in complex model systems. Uwamori et al., in a microfluidic model similar to ours, compared HUVECs with brain microvascular ECs (BMECs) and found that HUVECs formed more extensive microvascular networks, emphasizing their robust angiogenic potential.

Thus, HUVECs were chosen in our studies for their well-established nature as a widely used endothelial cell line for microvasculature in vitro formation. Also, they exhibit robust proliferation, are easily accessible, and can be expanded more readily than certain microvascular endothelial cells. This practicality facilitates experimental procedures and ensures an ample cell supply for our microfluidic model.

While acknowledging the distinct characteristics of organ-specific microvascular cells, as highlighted by Marcu et al. [35], it is worth noting that commercially available human microvascular endothelial cells are limited in terms of sources (i.e., bladder, heart skin, lung and myometrium). We were constrained by the unavailability of specific microvascular cells from the neuroblastoma niche or the adrenal gland, a common site for neuroblastoma development. In light of this limitation, HUVECs emerged as a practical compromise, allowing us to capture essential aspects of neuroblastoma angiogenesis and vasculature dynamics.

NB cells cultured with HUVEC in the presence of EGM did not increase cell death in 2D. A similar phenomenon is also observed when adipose-derived stem cells (ASCs) are co-culture with HUVEC [36]. ASCs do not differentiate into endothelial cells in the presence of EGM but protect HUVECs from death, which ratifies the necessary interconnection and support of the different cells in the tissue microenvironment. However, experiments in 3D of co-cultures of NB and HUVEC in EGM medium demonstrated the effect of dimensionality on cells’ fate. HUVECs seemed to protect NB from the adverse EGM effects in 2D, but we observed relevant cell death in 3D at 48h. The occurrence of cell death can be linked to the system being in a static state. Static cultures in 3D tend to create a necrotic core. They mimic oxygen and nutrient diffusion so that cells receive less oxygen and growth factors from the medium than in traditional 2D cultures [37]. For this reason, and depending on the type of tumor and the biological feature to recapitulate, it is necessary to include perfusion in the system.

In recent years, tumor-on-a-chip models have gained significant attention in biomedical research and drug development due to their ability to provide more physiologically relevant insights into tissue and organ behavior than traditional 2D cell cultures.

When constructing a tumor-on-a-chip, careful consideration of the initial cell number for cultivation within the chip is paramount. In the context of cancer, where rapid cell proliferation is a characteristic hallmark, understanding the dynamics of cancer cell cultures is crucial.

In our experimental design, we aimed to replicate and partially re-establish the distinctive phenotype and features observed in patients, a process demanding a minimum of 1 week of culture within the chip. Initiating the culture with a hydrogel densely populated with cells could lead to the formation of an overcrowded tissue within the central chamber of the gel after 1 week. This scenario poses the risk of inducing cell death and, in extreme cases, compromising the integrity of the chip due to increased pressure generated by the tissue. Therefore, the intentionally low initial cell density used in our protocol allowed for controlled and optimized cell proliferation, ensuring a more sustainable and viable tissue over the course of the experiment.

Vascularized tissue–engineered models are being built to offer valuable insights into cancer biology and potential treatments [38]. Because the vasculature influences drug delivery to the tumor site, vascularization is vital for drug testing and treatment development. Also, recapitulating vascularization in these models holds significant importance for studying the mechanisms of cancer cell intravasation and extravasation, providing insights into the metastatic process and potential targets for intervention. The complexity of the tumor microenvironment is another reason for including vascularization in in vitro models [38].

Our study demonstrated the significance of shear stress in promoting vascularization within the FX5 biomaterial. Shear stress is a fundamental biomechanical force that endothelial cells experience in the bloodstream, and its role in inducing vasculature formation is well-documented [39]. In the context of tumoral vessels potentially experiencing distinct shear stress values from healthy vessels, our decision to employ a wall shear stress of 36 dynes/cm^2^ (143 μl/min) was guided by the established range observed in healthy vessels, and the absence of specific data on shear stress in neuroblastoma capillaries. Also, the selection of a “healthy” stress value was sustained in that fact that transdifferentiation occurs in the context of pre-existing “healthy” capillaries from the host [40]. This observation aligns with prior published work [14, 26], where TECs coexisted with “healthy” endothelial cells in capillaries from patients’ tumors [26] Subsequently, we devised a model employing a self-setting strategy for capillary formation through the co-culture of endothelial cells and neuroblastoma cells, necessitating an initial shear stress. The chosen shear stress value serves as a foundational approach for simulating vascularization in a healthy context, representing a crucial precursor for the subsequent transdifferentiation processes in neuroblastoma. The successful application of “healthy” shear stress in the NB-on-a-chip model highlights its potential for simulating physiological conditions and improving the accuracy of the NB microenvironment representation.

Neuroblastoma tumors, like other tumors, encompass a small population of cancer stem cells capable of transdifferentiating into an even smaller population of TECs [40, 41] As illustrated in real tumors [14, 26], the presence of TECs is confined to a limited number of cells, aligning with observations in our in vitro model, a phenomenon not previously recapitulated in a TOAC model.

In conclusion, while acknowledging that the level of complexity in our model may not fully mirror patients’ tumors, our NB-on-a-chip model serves as a valuable tool to explore specific aspects of neuroblastoma vascularization and transdifferentiation, offering insights into the dynamic interplay between tumor cells and the endothelial microenvironment. The selection of FX5 as an appropriate biomaterial and the introduction of shear stress through a perfusion system allowed viable co-cultures and enhanced vascularization. The identification of tumor-derived endothelial cells with dual marker expression confirms the existence of an alternative vasculature in the neuroblastoma model. The model system developed can pave the way for innovative approaches to exploring therapeutic strategies that target both tumor cells and vasculature. In future studies, we hope to include microvascular cells from a relevant organ source to refine our model further. Although, as mentioned before, obtaining neuroblastoma organ–specific microvascular cells is a real challenge. Alternatively, we will explore the use of iPSC-ECs. We aim to include iPSC-ECs derived from patients in our chip, which presents a promising avenue for personalized therapies, albeit with the recognition of their reduced capillary network formation compared to HUVECs, as demonstrated in prior studies [42].

## Conclusions

This research addresses the challenge of mimicking the neuroblastoma microenvironment by developing biomaterials with appropriate stiffness. FX5 proved to be the most suitable formulation, supporting viable co-cultures and vessel-like structures. Introducing shear stress led to enhanced vascularization, culminating in the identification of tumor-derived endothelial cells expressing both CD31 and MYCN markers. These findings provide valuable insights into recreating neuroblastoma’s alternative vasculature and contribute to developing more accurate and predictive in vitro models for studying tumor behavior and exploring potential therapeutic interventions.

## Supplementary information

ESM 1 Supplementary Figure 1. Survival analysis of monocultures of neuroblastoma cells on FX5 and FX3 biomaterials with RPMI/EGM medium in 2D and under static conditions. Representative live/dead staining images of neuroblastoma cells with RPMI and endothelial medium (EGM) (1:1) at day 7 (n=3). Hoechst 33342, nuclei, blue; Calcein staining, green-live cells; ethidium homodimer-1 staining, red-dead cells. Top left - Brightfield images for each type of biomaterial assayed. Supplementary Figure 2. characterization of neuroblastoma and HUVEC co-cultures in 3D FX5 hydrogel in the microfluidic chip.(A) Merged live/dead fluorescence images of neuroblastoma and HUVEC co-cultures seeded in the 3-dimensional FX5 hydrogel loaded in the central chamber of the chip for 24 and 48 hours; 1000 cells/chip co-cultures (n=5 per time point). Hoechst staining (Hoechst 33342, nuclei; blue), calcein staining (green-live cells), ethidium homodimer-1 staining (red-dead cells). (B) Detail of a cell aggregate displaying live cells (left, calcein AM staining in green), dead cells (middle, ethidium homodimer-1 staining- EthD-1 in red), and a merge of both live/dead dyes (right). (C) Cell aggregate exhibiting cell sprouting (white arrows) at 48 hours (DOCX 3449 kb)

## Data Availability

The datasets during and/or analyzed during the current study are available from the corresponding author upon reasonable request.
