# Supplementary material for: Microfluidic model of the alternative vasculature in neuroblastoma
Source: In Vitro Model. 2024 Jan 15;3(1):49–63. doi: 10.1007/s44164-023-00064-x (PMC11756480; doi:10.1007/s44164-023-00064-x)
Supplement: Supplementary file 1 — Supplementary Figure 1. Survival analysis of monocultures of neuroblastoma cells on FX5 and FX3 biomaterials with RPMI/EGM medium in 2D and under static conditions. Representative live/dead staining images of neuroblastoma cells with RPMI and endothelial medium (EGM) (1:1) at day 7 (n=3). Hoechst 33342, nuclei, blue; Calcein staining, green-live cells; ethidium homodimer-1 staining, red-dead cells. Top left - Brightfield images for each type of biomaterial assayed. Supplementary Figure 2. characterization of neuroblastoma and HUVEC co-cultures in 3D FX5 hydrogel in the microfluidic chip.(A) Merged live/dead fluorescence images of neuroblastoma and HUVEC co-cultures seeded in the 3-dimensional FX5 hydrogel loaded in the central chamber of the chip for 24 and 48 hours; 1000 cells/chip co-cultures (n=5 per time point). Hoechst staining (Hoechst 33342, nuclei; blue), calcein staining (green-live cells), ethidium homodimer-1 staining (red-dead cells). (B) Detail of a cell aggregate displaying live cells (left, calcein AM staining in green), dead cells (middle, ethidium homodimer-1 staining- EthD-1 in red), and a merge of both live/dead dyes (right). (C) Cell aggregate exhibiting cell sprouting (white arrows) at 48 hours (DOCX 3449 kb) [file 44164_2023_64_MOESM1_ESM.docx]

**Supplementary Figure 1. Survival analysis of monocultures of neuroblastoma cells on FX5 and FX3 biomaterials with RPMI/EGM medium in 2D and under static conditions.** Representative live/dead staining images of neuroblastoma cells with RPMI and endothelial medium (EGM) (1:1) at day 7 (n=3). Hoechst 33342, nuclei, blue; Calcein staining, green-live cells; ethidium homodimer-1 staining, red-dead cells. Top left - Brightfield images for each type of biomaterial assayed.

**Supplementary Figure** **2. characterization of neuroblastoma and HUVEC co-cultures in 3D FX5 hydrogel in the microfluidic chip.(A)** Merged live/dead fluorescence images of neuroblastoma and HUVEC co-cultures seeded in the 3-dimensional FX5 hydrogel loaded in the central chamber of the chip for 24 and 48 hours; 1000 cells/chip co-cultures (n=5 per time point). Hoechst staining (Hoechst 33342, nuclei; blue), calcein staining (green-live cells), ethidium homodimer-1 staining (red-dead cells). (**B)** Detail of a cell aggregate displaying live cells (left, calcein AM staining in green), dead cells (middle, ethidium homodimer-1 staining- EthD-1 in red), and a merge of both live/dead dyes (right). **(C)** Cell aggregate exhibiting cell sprouting (white arrows) at 48 hours
